# American Veterinary Medical Association

**Published:** 1901-07

**Authors:** S. Stewart

**Affiliations:** Secretary; Kansas City, Mo.


### COMMUNICATION.

### AMERICAN VETERINARY MEDICAL ASSOCIATION.

The following circular letter has been issued by the active and energetic Secretary, Dr. S. Stewart, 1404 Holmes Street, Kansas City, Mo.:

KANSAS CITY, MO., June 1, 1901.

DEAR DOCTOR: The A. V. M. A. will hold its thirty-eighth annual meeting at Atlantic City, N. J., on September 3, 4, and 5, 1901. The selection of Atlantic City as the place for holding this meeting must be a pleasing one to the members, because of the social privileges it affords. The veterinarians of New Jersey, fully realizing that the influence of this Association enlarges public knowledge and appreciation of veterinary science, particularly in the regions where its meetings are held, extended a unanimous invitation to this Association to come to New Jersey. While it is true that the influence of the Association is a powerful factor in stimulating local professional good-will, professional enthusiasm, and public confidence, this influence spreads over all the area from which the Association draws its membership. Yes, far beyond, because the papers presented and discussed at its meetings are published in an annual report in the veterinary journals and in part in the public press; thus the veterinarians and the general public everywhere are given a broader view of the veterinary profession and its wide sphere of usefulness.

The number in attendance at the meeting in Detroit last year exceeded that of any previous meeting. About fifty ladies accompanied the members and visitors, and they participated in and added greatly to the social pleasures of the meeting. The programme for the meeting to be held in Atlantic City will be a very attractive one, and it is confidently expected that the attendance will be even larger than last year. Who can afford to stay away if it is possible to attend? This timely notice is given that you may plan to be present.

The difficulties encountered in the enforcement of municipal and sanitary regulations must always engage the serious attention of veterinarians. A constantly increasing number of them are employed in the control of tuberculosis, glanders, hog-cholera, and other diseases, slaughter-house and market inspection, milk and dairy inspection. New problems and new phases of old problems are constantly developing. The programme of the coming meeting will contain a number of papers relating to sanitary medicine.

The clinics held in connection with the last few meetings have proven so interesting and instructive that this feature of the programme will be given more time during the coming meeting. It is probable that one day will be devoted to clinics, and that a large number of operations will be demonstrated. Prominence will be given to the demonstration of the technique of the application of aseptic and antiseptic measures in some of the more common surgical operations. Well-known surgeons have promised to conduct the clinic. Facilities will be provided for all present to see the operations and to hear the remarks made by the operators. The explanatory remarks made in connection with each surgical operation and the numerous papers relating to general practice must certainly appeal very strongly to every practising veterinarian.

The committee on local arrangements has its plans about completed, and you are assured that nothing will be lacking to make the social phase of our meeting a most pleasant one. Atlantic City is famous as a seaside pleasure resort, and members and visitors who attend this meeting of the A. V. M. A. will certainly join the hosts who sing its praises as a grand meeting-place in which to have a good time.

If you intend to prepare a paper for the programme of the Atlantic City meeting, and you are respectfully urged to do so, you should notify me at once, stating title of same.

If there be any errors in the address on the enclosed envelope, kindly advise me of the same that our books may be corrected and make more certain the delivery of future communications from this office.

If your neighbor veterinarian is likely to be interested in the work of this Association and would probably apply for membership if solicited, will you not write to the resident secretary of your State and secure an application blank and copy of By-Laws for your neighbor's consideration?

Your attention is respectfully called to the enclosed statement of your account for dues, and you are urged to give this matter early attention. You know upon reflection that prompt payment of dues constitutes an elixir of life to an organization. Your proportion of said elixir is needed to maintain the vitality of the A. V. M. A.

Yours very sincerely,

S. Stewart,
Secretary.

